# Stroke mortality negatively associated with health, education, and safety: an ecological study, Minas Gerais, 2014-2022

**DOI:** 10.1590/S2237-96222025v34e20240820.en

**Published:** 2025-09-29

**Authors:** Daniel Hideki Bando, Francisco Chiaravalloti, Alfredo Pereira de Queiroz

**Affiliations:** 1 Universidade Federal de Alfenas, Instituto de Ciências da Natureza, Alfenas, MG, Brazil; 2 Universidade de São Paulo, Faculdade de Saúde Pública, São Paulo, SP, Brazil; 3 Universidade de São Paulo, Faculdade de Filosofia Letras e Ciências Humanas, São Paulo, SP, Brazil

**Keywords:** Stroke, Mortality Registries, Spatio-Temporal Analysis, Spatial Analysis, Ecological Studies, Accidente cerebrovascular, Registros de mortalidad, Análisis espacio-temporal, Análisis espacial, Estudios ecológicos

## Abstract

**Objectives:**

To analyze the spatial-temporal pattern of stroke mortality in the Brazilian state of Minas Gerais between 2014 and 2022 and to identify its association with socioeconomic indicators. The identification of clusters of high and low mortality rates, along with their associated indicators, may assist public managers in formulating intersectoral public policies aimed at reducing social inequalities.

**Methods:**

: This study employed an ecological approach using municipal-level data. Mortality data were obtained from the Department of Information Technology of the Brazilian National Health System. The covariates were the five dimensions that compose the Minas Gerais Social Responsibility Index developed by the João Pinheiro Foundation. Scan statistics were used to detect spatial and spatio-temporal clusters of high and low stroke mortality rates. The association between stroke mortality and the indicators was estimated using a spatial autoregressive model.

**Results:**

A total of 88,429 deaths due to stroke occurred during the study period, corresponding to a rate of 47.0 per 100,000 inhabitants per year. Purely spatial analysis identified 13 dispersed clusters: eight with high mortality rates (relative risk [RR] 1.08 to 1.31) and five with low rates (RR 0.80 to 0.89). Spatial-temporal analysis identified four high-rate clusters (2014-2017; RR 1.18 to 1.48) and three low-rate clusters (2018-2022; RR 0.66 to 0.87) distributed across the state. A negative association was found between stroke mortality and the indicators for health (β = -7.64), education (β = -7.21), and safety (β = -3.43), with R² = 0.41.

**Conclusion:**

This study can support public managers in monitoring, evaluating, and formulating public health policies by promoting articulation between health and other sectors, aiming to improve the population’s social well-being.

## Resumen

**Objetivos:**

Analizar el patrón espacio-temporal de la mortalidad por accidente cerebrovascular (ACV) en Minas Gerais entre 2014 y 2022 e identificar su asociación con indicadores socioeconómicos. La identificación de conglomerados de tasas altas y bajas y de indicadores asociados puede ayudar a los gestores a formular políticas públicas intersectoriales destinadas a reducir las desigualdades sociales.

**Métodos:**

Se trata de un estudio ecológico con datos municipales. Se utilizaron datos de mortalidad del Departamento de Información e Informática del Sistema Único de Salud. Las covariables fueron las cinco dimensiones que componen el índice de responsabilidad social de Minas Gerais de la Fundación João Pinheiro. Se utilizó una estadística de barrido para detectar conglomerados espaciales y espacio-temporales de tasas altas y bajas de mortalidad por ACV. La asociación entre el ACV y los indicadores se estimó mediante el modelo autorregresivo espacial.

**Resultados:**

Se produjeron 88 429 muertes por ACV (tasa de 47,0/100 000 habitantes por año). El análisis puramente espacial identificó 13 conglomerados dispersos, ocho de ellos con tasas altas (riesgo relativo [RR] 1,08 a 1,31) y cinco con tasas bajas (RR 0,80 a 0,89). El análisis espacio-temporal identificó cuatro conglomerados de tasas altas (2014-2017; RR 1,18 a 1,48) y tres de tasas bajas (2018-2022; RR 0,66 a 0,87) dispersos en el estado. Se identificó una asociación negativa entre la mortalidad por ACV y los indicadores de salud (β=-7,64), educación (β=-7,21), seguridad (β=-3,43) y R^2^ 0,41.

**Conclusión:**

Este estudio puede servir de base a los gestores para el seguimiento, la evaluación y la formulación de políticas públicas en el ámbito de la salud, con el fin de articularlas con otros sectores, en pro del bienestar social de la población.

**Ethical aspects**

This research used public domain anonymized databases.

## Introduction

Stroke was the third leading cause of death and the fourth leading cause of disability worldwide in 2021 ^1^. In Brazil, in 2022, stroke was the third leading cause of death, with 120,658 deaths, representing 7.8% of total deaths ^2^. In 2021, the age-adjusted global mortality rate from stroke was 87.5/100,000 inhabitants. 

According to the regions defined by the World Health Organization, Africa had the highest rate (122.8/100,000), while the Americas had the lowest rate (38.1/100,000). In Brazil, the rate was 51.8/100,000, ranking 152nd in the world. There were variations between states, with the lowest rate in Rondônia (35.6/100,000) and the highest in Maranhão (89.9/100,000). In Minas Gerais, the rate was 49.1/100,000 ^1^. Stroke mortality was analyzed spatially at the municipal level in Brazilian states, such as Rio de Janeiro ^3^, Ceará ^4^, and Alagoas ^5^. In the case of Alagoas, the elderly population was considered, and the local Moran index identified a cluster of high rates in the east and a cluster of low rates in the west ^5^. 

In Minas Gerais, based on micro-region data and an investigation into the spatial-temporal evolution of stroke mortality from 1980 to 2021, a downward trend was identified throughout the period. From 2015 to 2021, some micro-regions in the Northeast, such as Governador Valadares, Teófilo Otoni, and Nanuque, had the highest rates, ranging from 41.0 to 44.0 per 100,000 inhabitants ^6^. However, rates by micro-region may mask municipal inequalities.

Modifiable risk factors for stroke mortality were estimated using data from the 2017 Global Burden of Disease study, which included data from Brazil. In descending order of importance, the factors were: hypertension, diabetes, unhealthy eating habits, obesity, smoking, air pollution, alcohol use, hypercholesterolemia, and physical inactivity. Almost half of mortality was related to these factors and was therefore preventable ^7^. 

In Brazil, based on state-level data from 2010, a negative association was identified between stroke mortality and the Human Development Index (HDI) and years of schooling ^8^. Based on information from Brazilian municipalities, the impact of the Family Health Strategy on reducing the risk of stroke mortality was identified ^9^. Similarly, using municipal data, a higher risk of death from stroke was identified where there was a greater supply of ultra-processed foods ^10^. 

Understanding the spatial-temporal patterns of stroke mortality, as well as identifying associated risk factors at the municipal level, is crucial for developing effective public policies, including prevention programs, access to services, and health promotion initiatives. Furthermore, these analyses can be replicated in different populations, suggesting new hypotheses.

This study aimed to analyze the spatial-temporal pattern of stroke mortality and to identify its association with socioeconomic indicators, using municipal data from Minas Gerais between 2014 and 2022.

## Methods

Design

This was an ecological study in which the units of analysis were municipalities. The study period was 2014-2022. Social indicators were available for the years 2016, 2018, and 2020. The year 2018 was chosen as it represented the midpoint of the mortality and social indicator data.

Setting

**Figure 1:**
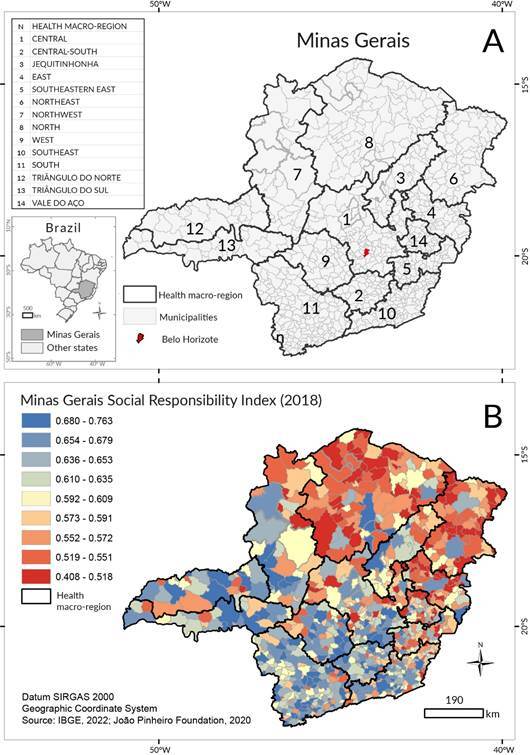
Location of health macro-regions (A) and Minas Gerais social responsibility index (B). Minas Gerais

In 2022, Minas Gerais had a population of 20.5 million, making it the second most populous state in Brazil ^11^. The capital, Belo Horizonte, was the most populous city, with 2.3 million inhabitants, while the median municipality was Araponga, with 8,048 inhabitants. Approximately 28.9% of municipalities had fewer than 5,000 inhabitants ^11^. A total of 853 municipalities were grouped into 14 health macro-regions (Figure 1A). 

In 2021, the Human Development Index (HDI) of Minas Gerais was 0.774, the fourth highest in Brazil ^11^. The Social Responsibility Index of Minas Gerais (IMRS), developed by the João Pinheiro Foundation, included all municipalities in the state ^12^. The IMRS is a synthetic social indicator, similar to the HDI, composed of 32 variables that range from zero (worst condition) to one (best condition). It is the weighted average of five dimensions: health, education, public safety, vulnerability, and sanitation. Each dimension includes a set number of indicators, all ranging from zero to one:

Health: composed of eight indicators (e.g., vaccination coverage, population covered by the Family Health Strategy). 

Education: composed of eight indicators (e.g., complete elementary school, age-grade distortion rate). 

Public safety: composed of three indicators (homicide rate, rate of crimes against property, and inhabitants per military police officer). 

Vulnerability: composed of ten indicators (e.g., poverty, Bolsa Família beneficiaries).

Sanitation: composed of six indicators (e.g., water supply, sewage services). 

The spatial distribution of the IMRS was used to describe the socioeconomic conditions in Minas Gerais. The highest scores were found in municipalities in the southwestern and southern regions, while the lowest scores were observed in the northern and northeastern regions (Figure 1B). 

Variables and data sources

All stroke-related deaths were classified by the following variables: sex (male, female); age (in years: ≤19, 20-39, 40-49, 50-59, 60-69, 70-79, ≥80); municipality of residence; and year of death (2014-2022).

Of the total 88,494 deaths recorded during the study period, 34 (0.038%) had no information on municipality of residence and were therefore excluded. Mortality data were obtained from the Mortality Information System (SIM) of the Brazilian Ministry of Health ^2^. Stroke deaths were defined as those coded I60 to I69 in the International Statistical Classification of Diseases and Related Health Problems 10th edition (ICD-10) ^2^. Population data were drawn from demographic censuses and respective projections for the intercensal periods, from the same website. The cartographic base of the study consisted of the territorial mesh of Minas Gerais, by municipality ^11^.

Statistical methods

Spatial-temporal cluster detection was performed using scan statistics with the SaTScan software ^13^. This method places a circular window of varying size over the centroid of each area; the radius increases to sweep in and add neighboring centroids. The maximum cluster size adopted was 15.0% of the population, defined by the Gini coefficient cluster reporting option. This function uses the Gini coefficient to optimize the selection of clusters, prioritizing those that best reflect differences in mortality risk among areas. 

Under the null hypothesis, the relative risk (RR) of the cluster was calculated and assessed for statistical significance using the likelihood ratio test with Monte Carlo simulations. The variables “age group” and “sex” were included as adjustment variables. Three types of analysis were conducted: purely temporal, purely spatial, and spatial-temporal. SaTScan classified clusters as high-rate when the relative risk (RR) was significantly greater than 1, and as low-rate when the RR was less than 1. The spatial-temporal analysis identified periods during which the clusters’ RRs were significant; the purely temporal analysis identified periods with significant RR values, either above or below 1. The significance level adopted was 5%. Maps were produced using ArcGIS software.

The association between stroke mortality and social indicators was estimated using regression models. The dependent variable, stroke mortality rate, was age-adjusted using the direct method and the World Health Organization’s standard population ^14^. To minimize rate instability and random fluctuation in municipalities with small populations, local empirical Bayesian smoothing was applied. This technique calculates a weighted average between the rate of each area and that of neighboring areas, with weights proportional to the underlying population at risk in each area. Areas with smaller populations tend to have substantially adjusted rates, whereas in areas with larger populations, the rates remain relatively unchanged ^15^. 

The independent variables were the five IMRS dimensions: health, education, public safety, vulnerability, and sanitation. Multiple linear regression and spatial autoregressive models were employed for testing. The latter accounted for the spatial dependence of the dependent variable through a spatial autoregressive parameter. Regression models were run in GeoDa software. Multicollinearity was assessed using the variance inflation factor for the variables. Moran’s I global spatial autocorrelation was used to assess the spatial autocorrelation of the residuals. Residual normality was checked using a histogram. Homoscedasticity was evaluated using scatter plots of residuals versus predicted values and versus each variable in the final model. The final model was chosen based on residual analysis, R², and the Akaike Information Criterion, which considers both the model’s fit and complexity.

## Results

**Figure 2.**
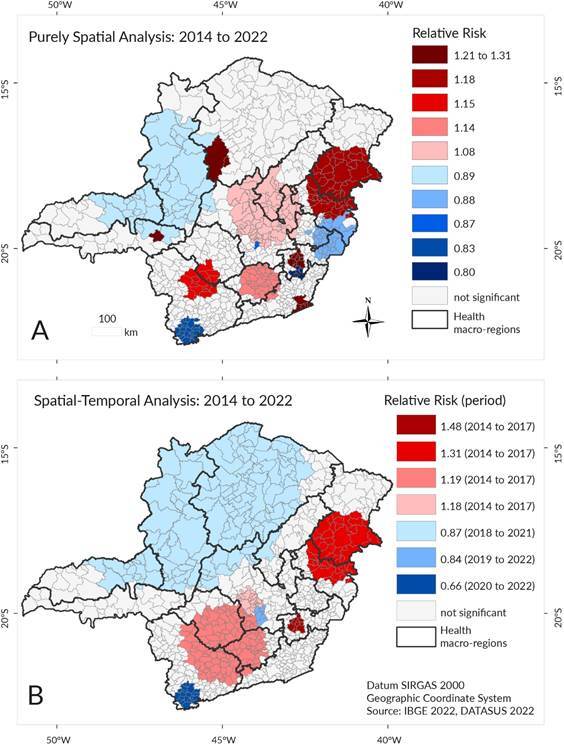
Spatial scan test of mortality from stroke: (A) Purely spatial analysis and (B) Spatial-temporal analysis. Minas Gerais, 2014-2022

.0 per 100,000 inhabitants per year. The purely temporal analysis detected one cluster in the 2014-2017 period, with a relative risk (RR) of 1.17, and indicated a decreasing trend in rates. The purely spatial analysis identified 13 clusters, comprising eight with high rates and five with low rates, dispersed across the state (Figure 2A). Buritizeiro and Pirapora in the Northern region (RR 1.31) and Araxá in the Southern Triangle macro-region (RR 1.23) stood out. In these areas, the risk of death from stroke was up to 31.0% higher than in areas outside the cluster. A cluster of 54 municipalities with an RR of 1.18 was located in the Eastern and Northeastern macro-regions. In the Far South, a cluster with an RR of 0.83 was identified, indicating a 17.0% lower risk of death from stroke. Four high-rate clusters bordered low-rate clusters. The capital, Belo Horizonte, had an RR of 0.88.

The spatial-temporal analysis identified high-rate clusters at the beginning of the study period (2014-2017), while low-rate clusters were observed at the end (2018-2022) (Figure 2B). The cluster with the highest RR (1.48) comprised 10 municipalities in the Southeastern macro-region, where the risk of stroke mortality was 48.0% higher. The cluster with an RR of 1.31 was composed of nearly the same municipalities as in the purely spatial analysis. Another notable cluster (RR 1.18) consisted of 136 municipalities in the Southern, South-Central, and Western macro-regions. As for the low-rate clusters, three were identified: the smallest (RR 0.84), which included the capital and bordered two high-rate clusters; the second, located in the Far South (RR 0.66); and the largest, located in the Northwest (RR 0.87).

**Figure 3.**
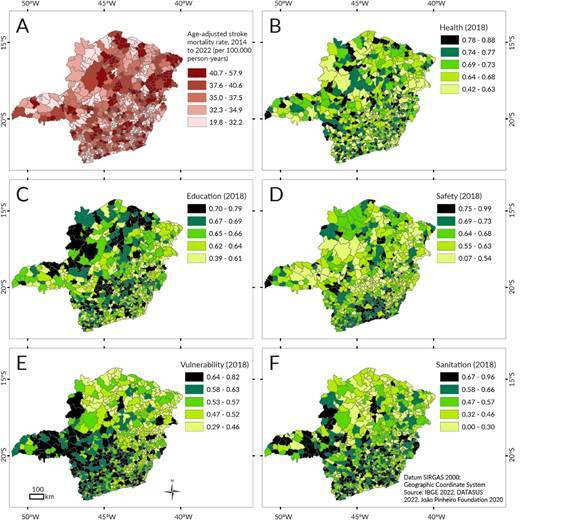
Indicators used in the regression model: (A) stroke mortality rate. Dimensions of the Minas Gerais social responsibility index: (B) health, (C) education, (D) safety, (E) vulnerability, (F) sanitation

The following series of maps illustrates the stroke mortality rates and the social indicators used in the association test. The spatial pattern was not clearly defined, as high rates were found in the municipalities of the Northeastern health macro-region and scattered nuclei across the state (Figure 3A). Some independent variables presented a more defined pattern, such as vulnerability and sanitation (Figures 3E and 3F), with the highest values concentrated in the Southern and Southeastern Regions. The multiple linear regression model showed a significant negative association between stroke mortality and three indicators. However, the residuals exhibited spatial dependence (Table 1), making the model unsuitable. The final model adopted was the spatial regression model, in which a spatial autoregressive parameter was incorporated to represent the spatial dependence of stroke mortality rates not explained by the covariates considered. In this model, the health, education, and public safety indicators showed significant negative associations with stroke mortality. Health had the greatest effect, followed by education and public safety (Table 1). For the health indicator, a one-unit increase, corresponding to the maximum index value, was associated with a decrease of 7.6 stroke deaths per 100,000 inhabitants. The final model yielded an R² of 0.41, surpassing the multiple linear regression model and exhibiting a lower Akaike Information Criterion, indicating a better fit. The residuals showed no spatial dependence (Table 1). 

**Table 1.** Variables associated with the stroke mortality rate. Minas Gerais, 2014-2022

	Linear regression		Spatial autoregressive model	
	Coefficient	p-value	Coefficient	p-value
Intercept	57.51	<0.001	23.35	<0.001
Health	-11.79	<0.001	-7.64	<0.001
Education	-15.62	<0.001	-7.21	0.014
Safety	-4.18	0.004	-3.43	0.003
Spatial autoregressive parameter (ρ)	-	-	0.694	<0.001
Residual I^a^	0.38	<0.001	0.002	0.200
R²	0.06	-	0.41	-
AIC^b^	5259.88	-	4964.86	-

^a^ Global Moran’s I of residuals.; ^b^Akaike Information Criterion.

**Figure 4:**
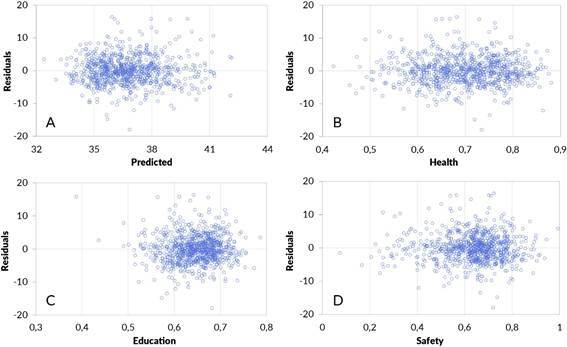
Residuals and predicted values (A), residuals and health (B), residuals and education (C), residuals and public safety (D).

The variance inflation factor was below 1.1 for all three variables, indicating no multicollinearity. The histogram of residual values showed a normal distribution. Scatter plots of residuals versus predicted values and versus each indicator showed no signs of heteroscedasticity (Figure 4).

## Discussion

Stroke mortality in Minas Gerais does not exhibit a well-defined spatial pattern, as clusters of both high and low rates were dispersed throughout the state. A similar pattern was observed in the states of Rio de Janeiro and Ceará; however, those studies did not apply specific techniques for cluster identification ^3^ ^,^ ^4^. Among older adults in Alagoas, from 2000 to 2016, Local Moran’s I identified a high-high cluster in the eastern region, including Maceió, and a low-low cluster in the western region ^5^. 

In the state of Rio de Janeiro, municipalities were grouped into health regions, except for the capital and Niterói. Stroke mortality was mapped for three time periods: 1979-1989, 1990-1999, and 2000-2010. In the latter two periods, the metropolitan region (excluding the capital and Niterói) and the northern region showed the highest rates. The capital and Niterói consistently had the lowest rates throughout the entire period. Better socioeconomic conditions may be associated with lower mortality rates ^3^. 

The pattern observed in Minas Gerais was similar, as Belo Horizonte was identified as a low-rate cluster and bordered high-rate clusters in the Central macro-region. This study identified a low-rate cluster in the Far South, characterized by the lowest RR, suggesting a possible relationship with the state of São Paulo. In 2016, cardiovascular disease mortality rates were mapped in the state of São Paulo. The lowest risk of death was found in areas bordering the far south of Minas Gerais, while the highest risk was observed in the southwest and northwest regions of São Paulo ^(16)^. Clarifying this issue will require further investigation. Future studies should be conducted to address this question. 

Identifying high- and low-rate mortality clusters is a key component for monitoring, evaluation, and the formulation of public health policies and should be considered alongside other indicators such as the prevalence and incidence of stroke ^(17)^. However, such data remain scarce at the municipal level. 

Using data from the Hospital Information System of Minas Gerais for 2019, the stroke hospitalization rate was found to be low in the Triângulo and Northwest macro-regions (up to 88.3 per 100,000 hospitalizations) and high in the Northeastern macro-region (ranging from 143.3 to 226.3 per 100,000). This same analysis also estimated the odds ratio (OR) for stroke patients being hospitalized outside their municipality of residence. In most of the state, the OR was greater than one, but it was lower (OR<1) in the Triângulo, Central (including the capital and surrounding areas), and Southern macro-regions ^18^. Such information is important for organizing the Brazilian National Health System (SUS) within the framework of healthcare networks, whose goal is to ensure universal and comprehensive access to health services efficiently and effectively ^19^ ^,^ ^20^. As for local initiatives, since 2020, the Minas Gerais State Health Department has enacted legislation with standards and procedures for organizing and operating the Stroke Care Line in the state, including accreditation and funding for reference hospitals to treat stroke patients ^21^. As of December 2024, Minas Gerais had 41 accredited stroke reference hospitals, the majority (eight) located in the Central macro-region ^22^. There were no reference hospitals in the Northeastern region, which, notably, had one of the highest rates of hospitalization for stroke ^18^. 

This study identified a negative association between stroke mortality and socioeconomic status, which was moderated by education, health, and public safety indicators. The vulnerability dimension was not included in the final model, despite encompassing social indicators such as poverty; this warrants further investigation in future studies. The negative association between stroke mortality and the education dimension in this study is consistent with previous literature ^8^ ^,^ ^24^ ^,^ ^25^ and may inform the development of intersectoral public policies. The education dimension, for example, includes indicators such as age-grade distortion rates and teacher training in basic education. Community-based education programs have also shown effectiveness in the primary prevention of stroke in low- and middle-income countries ^26^.

Most ecological studies in Brazil have shown a negative association between stroke mortality and socioeconomic conditions. One study found an association between income inequality and stroke mortality using state-level data from 2002 to 2009. A reduction of 0.1 in the Gini Index was associated with an 18.0% reduction in mortality rates ^27^. Another study identified a negative association between stroke mortality and both the Human Development Index (HDI) and average years of education, using state-level data from 2010 ^8^. Coverage by the Family Health Strategy was identified as a significant protective factor against stroke mortality (RR 0.82) in a study of 1,622 Brazilian municipalities from 2000 to 2009 ^9^. In 2016, municipal-level data from Brazil showed that greater availability of ultra-processed foods was associated with a 22.0% increase in stroke mortality risk ^10^. In Paraná, low education levels and residence in municipalities not hosting a Regional Health Office were identified as risk factors for stroke mortality in 2007 ^24^. 

In Rio de Janeiro, in 1991, 2000, and 2010, a negative association was identified between the HDI and stroke mortality using municipal data. An increase of 0.1 in HDI was associated with a reduction of 30.2 deaths per 100,000 inhabitants ^28^. In 23 municipalities of the Extended Health Region of Vale do Jequitinhonha (Minas Gerais), no association was found between stroke mortality and the HDI ^29^. Except for the last case, the findings of the present study are in agreement with those of previous studies; however, the socioeconomic indicators and association measures differ, limiting direct comparison.

Internationally, studies using aggregated data at the county, municipal, district, or census tract level have also found associations between stroke mortality and poorer socioeconomic conditions, consistent with these findings.

In Chile, a study using municipal data in 2003 found all stroke mortality risk factors to be statistically significant: diabetes (OR 1.25), physical inactivity (OR 1.13), self-perceived poor health (OR 1.11), obesity (OR 1.03), and poverty (OR 1.02). High socioeconomic status was a protective factor (OR 0.98) ^30^. In the United States, stroke mortality risk and protective factors were estimated using data from 3,226 counties. Lack of a college degree was a risk factor (OR 1.24), while higher median household income (OR 0.69) and access to parks (OR 0.85) were protective factors, all of which were statistically significant ^25^. In Madrid, Spain, using census tract data ^31^, and in Germany, using district data ^32^, higher stroke mortality risk was identified in areas with greater social deprivation. In Japan ^33^, Taiwan ^34^, and Australia ^35^, stroke mortality risk was associated with poorer socioeconomic conditions using municipal-level data.

This study had some limitations. One issue was the incomplete or incorrect completion of death certificates, which varied by region, and the possibility of underreporting in smaller municipalities ^24^. The quality of data in the Mortality Information System has improved in recent years, although there are regional differences. The proportion of deaths due to ill-defined causes (Chapter XVIII of ICD-10) in Minas Gerais decreased from 7.8% in 2014 to 6.9% in 2022. In the Northern macro-region, it fell from 16.5% in 2014 to 11.1% in 2022. In the Southern macro-region, the rate remained relatively stable, increasing from 4.9% in 2014 to 5.3% in 2022 ^2^. Data quality may have influenced the detection of high-rate clusters in the southern part of the state. Another limitation is inherent to the study design: it constitutes an ecological fallacy to attribute population-level estimates to individuals. 

Clusters of both high and low stroke mortality rates were dispersed across Minas Gerais. Stroke mortality was negatively associated with education, health, and public safety indicators. Identifying clusters and associated social indicators may support decision-makers in monitoring, evaluating, and developing public health policies, in coordination with other sectors such as education, to promote social well-being.
