# Supplementary material for: Stroke mortality negatively associated with health, education, and safety: an ecological study, Minas Gerais, 2014-2022
Source: Epidemiol Serv Saude. 2025 Sep 29;34:e20240820. doi: 10.1590/S2237-96222025v34e20240820.en (PMC12479032; doi:10.1590/S2237-96222025v34e20240820.en)
Supplement: Translation

# Translation: Mortalidade por acidente vascular cerebral associado negativamente com saúde, educação e segurança: estudo ecológico, Minas Gerais, 2014-2022

**DOI:** 10.1590/S2237-96222025v34e20240820.pt

**Authors:** Daniel Hideki Bando, Francisco Chiaravalloti, Alfredo Pereira de Queiroz

**Affiliations:** 1 Universidade Federal de Alfenas, Instituto de Ciências da Natureza, Alfenas, MG, Brasil; 2 Universidade de São Paulo, Faculdade de Saúde Pública, São Paulo, SP, Brasil; 3 Universidade de São Paulo, Faculdade de Filosofia Letras e Ciências Humanas, São Paulo, SP, Brasil

**Keywords:** Acidente Vascular Cerebral, Registros de Mortalidade, Análise Espaço-temporal, Análise Espacial, Estudos Ecológicos

## Abstract

**Objetivos:**

: Analisar o padrão espaço-temporal da mortalidade por acidente vascular cerebral (AVC) em Minas Gerais entre 2014 e 2022 e identificar sua respectiva associação com indicadores socioeconômicos. A identificação de conglomerados de taxas altas e baixas e indicadores associados pode auxiliar os gestores na formulação de políticas públicas intersetoriais, visando à redução das desigualdades sociais.

**Métodos:**

: Tratou-se de estudo ecológico, com dados municipais. Utilizaram-se dados de mortalidade do Departamento de Informação e Informática do Sistema Único de Saúde. As covariáveis foram as cinco dimensões que compõem o índice mineiro de responsabilidade social da Fundação João Pinheiro. Estatística de varredura foi usada para detectar conglomerados espaciais e espaço-temporais de taxas altas e taxas baixas de mortalidade por AVC. A associação entre o AVC e indicadores foi estimada pelo modelo autorregressivo espacial.

**Resultados:**

: Ocorreram 88.429 mortes por AVC (taxa de 47,0/100 mil habitantes por ano). A análise puramente espacial identificou 13 conglomerados dispersos, sendo oito de taxas altas (risco relativo [RR] 1,08 a 1,31) e cinco de taxas baixas (RR 0,80 a 0,89). A análise espaço-temporal identificou quatro conglomerados de taxas altas (2014-2017; RR 1,18 a 1,48) e três de taxas baixas (2018-2022; RR 0,66 a 0,87) dispersos no estado. Foi identificada associação negativa da mortalidade por AVC com os indicadores saúde (β=-7,64), educação (β=-7,21), segurança (β=-3,43) e R^2^ 0,41.

**Conclusão:**

: Este estudo pode subsidiar os gestores no monitoramento, na avaliação e na formulação de políticas públicas no campo da saúde, de modo a articular este com outros setores, visando ao bem-estar social da população.

**Aspectos éticos**

Esta pesquisa utilizou bancos de dados de domínio público e anonimizados.

## Introdução

O acidente vascular cerebral (AVC) foi a terceira principal causa de morte e a quarta principal causa de incapacidade no mundo em 2021 ^1^. No Brasil, em 2022, o AVC foi a terceira principal causa de morte, com 120.658 óbitos, o que representou 7,8% do total de óbitos ^2^. Em 2021, a taxa global de mortalidade por AVC ajustada por idade foi 87,5/100 mil habitantes. 

Considerando-se as regiões da Organização Mundial da Saúde, a africana apresentou a maior taxa (122,8/100 mil), e a região das Américas, a menor (38,1/100 mil). No Brasil, a taxa foi 51,8/100 mil, posição 152 no ranque mundial. Houve variações entre os estados, com a menor taxa em Rondônia (35,6/100 mil) e a maior no Maranhão (89,9/100 mil). Em Minas Gerais, a taxa foi 49,1/100 mil ^1^. Foi analisada espacialmente a mortalidade por AVC nos estados brasileiros, na escala municipal, como no Rio de Janeiro ^3^, no Ceará ^4^ e em Alagoas ^5^. No caso de Alagoas, foi considerada a população idosa, e o índice de Moran local identificou um conglomerado de taxas altas no Leste e um de taxas baixas no Oeste ^5^. 

Em Minas Gerais, utilizando-se dados de microrregiões e investigando-se a evolução espaço-temporal da mortalidade por AVC de 1980 a 2021, foi identificada tendência de redução em todo período. De 2015 a 2021, algumas microrregiões do Nordeste, como Governador Valadares, Teófilo Otoni e Nanuque, apresentaram as maiores taxas, de 41,0 a 44,0/100 mil habitantes ^6^. No entanto, as taxas por microrregiões podem encobrir desigualdades municipais.

Os fatores de risco modificáveis à mortalidade por AVC foram estimados com dados do projeto Carga de Doença Global de 2017, incluindo o Brasil, e, em ordem decrescente de importância, foram: hipertensão, diabetes, maus hábitos alimentares, obesidade, tabagismo, poluição do ar, alcoolismo, hipercolesterolemia e inatividade física. Quase metade da mortalidade estava relacionada a esses fatores, portanto preveníveis ^7^. No Brasil, utilizando-se dados estaduais de 2010, foi identificada associação negativa da mortalidade por AVC com o índice de desenvolvimento humano (IDH) e anos de estudo ^8^. Com base em informações de municípios brasileiros, foi identificado o impacto da cobertura da Estratégia Saúde da Família na redução do risco da mortalidade por AVC ^9^. Analogamente, também com dados municipais, foi identificado maior risco de morte por AVC onde houve maior oferta de alimentos ultraprocessados ^10^. 

Conhecer o padrão espaço-temporal da mortalidade por AVC, bem como identificar fatores de risco associados no nível municipal, é essencial para a formulação de políticas públicas, incluindo programas de prevenção, acesso aos serviços e promoção da saúde. Além disso, essas análises podem ser replicadas em diferentes populações e sugerir novas hipóteses.

Este estudo teve como objetivo analisar o padrão espaço-temporal da mortalidade por AVC e identificar sua respectiva associação com indicadores socioeconômicos, com dados municipais de Minas Gerais entre 2014 e 2022.

## Métodos 

Delineamento

Tratou-se de estudo ecológico cujas unidades de análise foram os municípios. O período escolhido foi 2014-2022. Os indicadores sociais estavam disponíveis nos anos 2016, 2018 e 2020. Foi escolhido o ano de 2018, por ser o meio do período da mortalidade e dos indicadores sociais.

Contexto

Em 2022, Minas Gerais tinha 20,5 milhões de habitantes e foi considerado o segundo estado brasileiro mais populoso ^11^. A capital Belo Horizonte foi a mais populosa com 2,3 milhões de habitantes, e a mediana foi Araponga com 8.048 habitantes. Cerca de 28,9% dos municípios apresentaram população inferior a 5 mil habitantes ^11^. Foram agrupados 853 municípios em 14 macrorregiões de saúde (Figura 1A). 

**Figura 1:** Localização das macrorregiões de saúde (A) e índice mineiro de responsabilidade social (B). Minas Gerais

Em 2021, o IDH de Minas Gerais foi 0,774, o quarto maior do Brasil ^11^. O índice mineiro de responsabilidade social (IMRS) da Fundação João Pinheiro abrangeu todos os municípios mineiros ^12^. Tratou-se de indicador social sintético, semelhante ao IDH, composto por 32 variáveis, o qual variou de zero (pior condição) a um (melhor condição). O IMRS foi a média ponderada de cinco dimensões: saúde, educação, segurança pública, vulnerabilidade e saneamento. Cada dimensão contemplou determinada quantidade de indicadores, conforme a seguir, os quais variaram de zero a um. 

Saúde: composta por oito indicadores (por exemplo, cobertura vacinal, população atendida pela Estratégia Saúde da Família). 

Educação: composta por oito indicadores (por exemplo, ensino fundamental completo, taxa de distorção idade-série). 

Segurança: composta por três indicadores (taxa de homicídio, de crimes contra o patrimônio e habitantes por policial militar). 

Vulnerabilidade: composta por dez indicadores (por exemplo, pobreza, beneficiários do Bolsa Família).

Saneamento: composta por seis indicadores (por exemplo, abastecimento de água, esgotamento). 

A distribuição espacial do IMRS foi usada para descrever as condições socioeconômicas de Minas Gerais. Os maiores índices se situaram nos municípios a sudoeste e sul, e os menores, no norte e nordeste (Figura 1B). 

Variáveis e fontes de dados

Todos os óbitos por AVC foram classificados pelas seguintes variáveis: sexo (homens, mulheres); idade (em anos: ≤19, 20-39, 40-49, 50-59, 60-69, 70-79, ≥80); local de residência do óbito (município); e ano do óbito (2014-2022).

No período de estudo, do total de 88.494 óbitos, 34 (0,038%) não tinham a informação do município de residência, portanto não foram usados. Os dados de mortalidade foram extraídos do Sistema de Informações sobre Mortalidade (SIM) do Ministério da Saúde ^2^. As mortes por AVC correspondem aos códigos I60 a I69 da décima Classificação Estatística Internacional de Doenças e Problemas Relacionados com a Saúde (CID-10) ^2^. Dados populacionais foram obtidos dos censos demográficos, com as respectivas projeções para os períodos intercensitários, na mesma página eletrônica. A base cartográfica do estudo constituiu-se da malha territorial de Minas Gerais, por municípios ^11^.

Métodos estatísticos

A detecção dos conglomerados espaço-temporais foi conduzida pela estatística de varredura, com o programa SaTScan ^13^. Essa estatística inseriu uma janela circular de tamanho variável no centroide de cada área; o raio aumentou de tamanho, varrendo e adicionando os centroides vizinhos. O tamanho máximo do conglomerado adotado foi 15,0% da população, definido pela opção de relatório de aglomerado de índice de Gini*.* Essa função utilizou o índice de Gini para otimizar a seleção de conglomerados, priorizando aqueles que melhor refletem as diferenças nos riscos de mortalidade entre as áreas. 

Sob a hipótese nula, calculou-se o risco relativo (RR) do conglomerado e verificou-se se o mesmo ocorreu ao acaso, utilizando-se para tanto o teste da razão de verossimilhança com base nas simulações de Monte Carlo. As variáveis “faixa etária” e “sexo” foram consideradas como variáveis de ajuste. Foram conduzidos três tipos de análise: puramente temporal, puramente espacial e espaço-temporal. O SaTScan considerou o conglomerado de taxas altas quando o RR foi significativamente maior do que 1 e de taxas baixas quando o RR foi inferior a 1. A análise espaço-temporal identificou em qual período os RR dos conglomerados foram significativos. A análise puramente temporal identificou em qual período ocorreu RR significativo, acima ou abaixo de um. O nível de significância adotado foi de 5%. Os mapas foram elaborados no programa ArcGIS.

A associação entre a mortalidade por AVC e os indicadores sociais foi estimada por modelos de regressão. A variável dependente, a taxa de mortalidade por AVC, foi ajustada por idade pelo método direto, com a população padrão da Organização Mundial da Saúde ^14^. Para minimizar a instabilidade das taxas e a flutuação aleatória dos municípios com populações pequenas, foi usada a taxa bayesiana empírica local. Essa técnica calculou a média ponderada entre a taxa de cada área e respectivas áreas vizinhas, com pesos proporcionais à população subjacente em risco. Áreas com populações pequenas tenderão a ter taxas consideravelmente ajustadas, enquanto, para populações maiores, as taxas mudarão pouco ^15^. 

As variáveis independentes foram as cinco dimensões do IMRS: saúde, educação, segurança, vulnerabilidade e saneamento. Foram testados a regressão linear múltipla e o modelo autorregressivo espacial. Este considerou a dependência espacial da variável dependente, incorporada pelo parâmetro espacial autorregressivo. Os modelos de regressão foram executados no programa GeoDa. A multicolinearidade foi verificada pelo fator de inflação da variância das variáveis. A autocorrelação espacial global de Moran foi usada para verificar a dependência espacial dos resíduos. A normalidade dos resíduos foi verificada por um histograma. A homocedasticidade foi avaliada por gráficos de dispersão dos resíduos em função dos valores preditos e de cada uma das variáveis do modelo final. A escolha do modelo final foi baseada na análise dos resíduos, no R**²** e no critério de informação de Akaike. Este considerou o ajuste e a complexidade do modelo.

## Resultados 

No período de estudo, ocorreram 88.429 mortes por AVC (taxa de 47,0/100 mil habitantes por ano). A análise puramente temporal detectou um conglomerado no período 2014-2017, com RR 1,17, e indicou tendência de diminuição das taxas. Na análise puramente espacial, foram detectados 13 conglomerados, sendo oito de taxas altas e cinco de taxas baixas dispersos pelo estado (Figura 2A). Destacaram-se Buritizeiro e Pirapora no Norte com RR 1,31 e Araxá na macrorregião Triângulo do Sul com RR 1,23. Nessas áreas, o risco de morte por AVC foi aumentado em até 31,0% em relação às áreas fora do conglomerado. Havia um conglomerado de 54 municípios com RR 1,18 situado nas macrorregiões Leste e Nordeste. No Extremo Sul, foi identificado um conglomerado com RR 0,83, portanto o risco de morte por AVC foi reduzido em 17,0%. Havia quatro conglomerados de taxas altas que faziam fronteira com conglomerados de taxas baixas. Belo Horizonte apresentou RR 0,88.

A análise espaço-temporal identificou conglomerados de taxas altas no início do período (2014-2017), já os de taxas baixas foram no final (2018-2022) (Figura 2B). O de maior RR (1,48) foi formado por 10 municípios e situava-se na macrorregião Leste do Sul, onde o risco de morte por AVC foi aumentado em 48,0%. O conglomerado com RR 1,31 foi formado praticamente pelos mesmos municípios da análise puramente espacial. Destacou-se também um conglomerado com RR 1,18 formado por 136 municípios nas macrorregiões Sul, Centro Sul e Oeste. Quanto aos conglomerados de taxas baixas, foram identificados três: o de menor área (RR 0,84) que incluiu a capital, a qual fazia fronteira com dois conglomerados de taxas altas; o segundo, no extremo sul (RR 0,66); e o de maior área, no noroeste (RR 0,87).

**Figura 2:** Teste de varredura espacial da mortalidade por acidente vascular cerebral: (A) análise puramente espacial e (B) análise espaço-temporal. Minas Gerais, 2014-2022

**Figura 3:** Indicadores usados no modelo de regressão: (A) taxa de mortalidade por acidente vascular cerebral. Dimensões do índice mineiro de responsabilidade social: (B) saúde, (C) educação, (D) segurança, (E) vulnerabilidade, (F) saneamento. Minas Gerais

A coleção de mapas a seguir representou as taxas de mortalidade por AVC e osindicadores sociais do teste de associação. Notou-se que o padrão espacial não é bem definido, visto que altas taxas foram encontradas nos municípios da macrorregião de saúde Nordeste e núcleos dispersos pelo estado (Figura 3A). Algumas variáveis independentes apresentaram padrão mais definido, por exemplo, vulnerabilidade e saneamento (Figuras 3E e 3F). Nesse caso, os maiores valores concentraram-se no Sul e no Sudeste. O modelo de regressão linear múltipla revelou associação negativa e significativa entre a mortalidade por AVC e três indicadores, porém os resíduos apresentaram dependência espacial (Tabela 1), portanto não foi apropriado. O modelo final adotado foi o de regressão espacial, no qual foi incorporado o parâmetro espacial autorregressivo, representando a dependência espacial das taxas de mortalidade por AVC não explicada pelas covariáveis consideradas. Nesse modelo, os indicadores saúde, educação e segurança apresentaram associação negativa e significativa com a mortalidade por AVC. Saúde apresentou maior efeito, seguido por educação e segurança (Tabela 1). No caso do indicador saúde, o aumento de uma unidade que seria o valor máximo do índice estava relacionado à diminuição da taxa de mortalidade em 7,6/100 mil habitantes. O modelo final apresentou R**²** 0,41, maior do que o modelo de regressão linear e com menor critério de informação de Akaike, conferindo melhor ajuste. Os resíduos não apresentaram dependência espacial (Tabela 1). 

**Tabela 1:** Variáveis associadas com a taxa de mortalidade por acidente vascular cerebral. Minas Gerais, 2014-2022

	Regressão linear		Modelo autorregressivo espacial	
	Coeficiente	p-valor	Coeficiente	p-valor
Intercepto	57,51	<0,001	23,35	<0,001
Saúde	-11,79	<0,001	-7,64	<0,001
Educação	-15,62	<0,001	-7,21	0,014
Segurança	-4,18	0,004	-3,43	0,003
Parâmetro espacial autorregressivo (ρ)	-	-	0,694	<0,001
Iresíduo^a^	0,38	<0,001	0,002	0,200
R²	0,06	-	0,41	-
AIC^b^	5259,88	-	4964,86	-

^a^ Índice de Moran global dos resíduos; ^b^Critério de informação de Akaike.

O fator de inflação da variância foi inferior a 1,1 para as três variáveis, o que indicou ausência de multicolinearidade. O histograma com os valores dos resíduos apresentou distribuição normal. Gráficos de dispersão dos resíduos com valores preditos, bem como dos resíduos e cada um dos indicadores, não apresentaram sinais de heterocedasticidade (Figura 4).

## Discussão

A mortalidade por AVC em Minas Gerais não tem padrão espacial definido, pois houve conglomerados de taxas altas e taxas baixas dispersos no estado. No estado do Rio de Janeiro e no Ceará, o padrão foi semelhante, no entanto não foram usadas técnicas para identificação de conglomerados ^3^ ^,^ ^4^. Em idosos de Alagoas, de 2000 a 2016, a estatística local de Moran identificou um conglomerado do tipo alto-alto a Leste, incluindo Maceió, e outro do tipo baixo-baixo a Oeste ^5^. 

No estado do Rio de Janeiro, os municípios foram agregados em regiões de saúde, com exceção da capital e de Niterói, e a mortalidade por AVC foi mapeada em três períodos: 1979-1989, 1990-1999 e 2000-2010. Nos dois últimos períodos, a região metropolitana (além da capital e de Niterói) e o Norte apresentaram as maiores taxas. A capital e Niterói apresentaram as menores taxas em todo período. Melhores condições socioeconômicas podem estar relacionadas com as menores taxas ^3^. 

**Figura 4:** Resíduos e valores preditos (A), resíduos e saúde (B), resíduos e educação (C), resíduos e segurança (D).

O padrão em Minas Gerais foi semelhante, dado que Belo Horizonte foi identificada como conglomerado de taxas baixas e fazia fronteira com conglomerados de taxas altas na macrorregião Centro. Este estudo identificou um conglomerado de taxas baixas no Extremo Sul, com o menor RR, e sugeriu uma possível relação com São Paulo. Foi mapeada a taxa de mortalidade por doenças cardiovasculares no estado de São Paulo em 2016. O risco de morte foi menor na área que fazia fronteira com o extremo sul de Minas Gerais e maior no sudoeste e no noroeste paulista ^16^. A elucidação dessa questão exige investigações adicionais. Novos estudos deverão ser conduzidos para esclarecer essa questão. 

A identificação dos conglomerados de taxas altas e baixas de mortalidade é um componente importante para o monitoramento, a avaliação e a formulação de políticas públicas de saúde e deve ser considerado junto com outros indicadores como a prevalência e incidência de AVC ^17^. No entanto, há carência dessas informações no nível municipal. 

Utilizando informações do Sistema de Informações Hospitalares de Minas Gerais referentes a 2019, observou-se que a taxa de internação por AVC era baixa nas macrorregiões de saúde Triângulo e Noroeste (até 88,3/100 mil internações), porém alta na macrorregião Nordeste (143,3 a 226,3/100 mil). Essa mesma análise também estimou a chance de deslocamento (*odds ratio*, OR) de pacientes internados por AVC para fora de seu município de residência. Na maior parte do estado, o OR foi maior do que um, sendo menor (OR<1) nas macrorregiões de saúde Triângulo, Centro (capital e adjacências) e Sul ^18^. São informações importantes para a organização do Sistema Único de Saúde, no âmbito das redes de atenção à saúde, cujo objetivo é garantir o acesso universal e integral aos serviços de saúde de maneira eficiente e eficaz ^19^ ^,^ ^20^. Quanto às iniciativas locais, a Secretaria de Estado de Saúde de Minas Gerais tem publicado legislações desde 2020, com normas e procedimentos para a organização e o funcionamento da Linha de Cuidado ao AVC no estado, o que inclui o credenciamento e o financiamento de hospitais de referência para atender pacientes com AVC ^21^. Em dezembro de 2024, Minas Gerais contava com 41 hospitais de referência para o tratamento de AVC, e a maioria (oito) estava localizada na macrorregião de saúde Centro ^22^. Não havia hospital de referência no Nordeste - vale destacar que essa macrorregião apresentou elevada razão de internação por AVC ^18^. 

Este estudo identificou associação negativa da mortalidade por AVC com nível socioeconômico, moderado pelos indicadores educação, saúde e segurança. A dimensão vulnerabilidade não entrou no modelo final, apesar de incluir indicadores sociais como a pobreza e isso pode ser investigado em estudos futuros. A associação da mortalidade por AVC com a dimensão educação neste estudo condiz com os achados da literatura ^8^ ^,^ ^24^ ^,^ ^25^ e pode fomentar a formulação de políticas públicas intersetoriais. A dimensão educação, por exemplo, inclui indicadores como taxa de distorção idade-série e formação docente da educação básica. Programas de educação com base na comunidade também têm demonstrado eficácia na prevenção primária do AVC em países de baixa e média renda ^26^.

A maioria dos estudos ecológicos do Brasil tem mostrado associação negativa da mortalidade por AVC com condições socioeconômicas. Foi identificada associação da desigualdade de renda com a mortalidade por AVC, com dados estaduais, de 2002 a 2009. A redução de 0,1 do índice de Gini foi associada à redução de 18,0% na taxa de mortalidade ^27^. Também foi estimada associação negativa da mortalidade por AVC com o IDH e a educação por anos de estudo, com dados estaduais de 2010 ^8^. A cobertura da Estratégia Saúde da Família foi identificada como fator de proteção significativo para mortalidade por AVC (RR 0,82), utilizando-se dados de 1.622 municípios brasileiros, de 2000 a 2009 ^9^. No Brasil, em 2016, dados de municípios apontaram que a maior disponibilidade de alimentos ultraprocessados esteve associada ao aumento de 22,0% no risco de morte por AVC ^10^. No Paraná, baixa escolaridade e residência em municípios não sede de Regional de Saúde foram identificados como fatores de risco de morte por AVC em 2007 ^24^. 

No Rio de Janeiro, em 1991, 2000, 2010, foi identificada associação negativa entre o IDH e a mortalidade por AVC, com dados municipais. O aumento de 0,1 do IDH foi associado à redução de 30,2 óbitos por 100 mil ^28^. Em 23 municípios da Região Ampliada de Saúde do Vale do Jequitinhonha (Minas Gerais), a mortalidade por AVC não foi associada ao IDH ^29^. Com exceção do último, os demais condizem com este estudo, no entanto os indicadores socioeconômicos e as medidas de associação variam e impedem comparação precisa.

O cenário internacional, de análises que usaram dados agregados por condados, municípios, distritos, setores censitários, tem mostrado associação da mortalidade por AVC com piores condições socioeconômicas, portanto condizem com este achado.

No Chile em 2003, utilizando-se dados municipais, foram elencados os fatores de risco à mortalidade por AVC, sendo todos significativos: diabetes (OR 1,25), sedentarismo (OR 1,13), percepção de saúde precária (OR 1,11), obesidade (OR 1,03) e pobreza (OR 1,02). Alto nível socioeconômico foi fator de proteção (OR 0,98) ^30^. Nos Estados Unidos, foram estimados os fatores de risco e proteção à mortalidade por AVC, com dados de 3.226 condados. Ausência de diploma universitário foi fator de risco (OR 1,24), já a renda mediana das famílias (OR 0,69) e o acesso a parques (OR 0,85) foram fatores de proteção, todos significativos ^25^. Em Madri, na Espanha, com dados por setores censitários ^31^, e na Alemanha, com dados por distritos ^32^, foi identificado maior risco de mortalidade por AVC em áreas de maior privação social. No Japão ^33^, em Taiwan ^34^ e na Austrália ^35^, foram estimadas associações do risco de morte por AVC com piores condições socioeconômicas, usando dados municipais.

Este estudo teve algumas limitações. Uma delas foi o preenchimento inadequado do atestado de óbito que pode variar de acordo com a região e a possibilidade de subnotificação em municípios pequenos ^24^. Houve melhora na qualidade da informação referente ao Sistema de Informações sobre Mortalidade nos últimos anos, com diferenças regionais. A proporção de óbitos por causas mal definidas (Capítulo XVIII da CID-10) em Minas Gerais foi 7,8% em 2014 e passou para 6,9% em 2022. Na macrorregião de saúde Norte, a proporção foi 16,5% em 2014 e 11,1% em 2022. Na macrorregião Sul, a proporção foi 4,9% em 2014 e 5,3% em 2022 ^2^. A qualidade da informação pode ter influenciado a detecção de agrupamentos de taxas altas no sul do estado. Outra limitação foi inerente ao desenho de estudo, visto que se denomina falácia ecológica atribuir ao indivíduo o resultado estimado em agregados populacionais. 

Havia conglomerados de taxas altas e taxas baixas de mortalidade por AVC dispersos em Minas Gerais. O AVC foi associado negativamente com os indicadores de educação, saúde e segurança. A identificação de conglomerados e indicadores sociais associados pode subsidiar os gestores no monitoramento, na avaliação e na formulação de políticas públicas no campo da saúde, de modo a articular este com outros setores, como a educação, visando ao bem-estar social.
